# Neuronal activity in posterior parietal cortex area LIP is not sufficient for saccadic eye movement production

**DOI:** 10.3389/fnint.2023.1251431

**Published:** 2023-11-24

**Authors:** Emiliano Brunamonti, Martin Paré

**Affiliations:** ^1^Department of Physiology and Pharmacology, Sapienza University of Rome, Rome, Italy; ^2^Department of Biomedical and Molecular Sciences, Queen’s University, Kingston, ON, Canada

**Keywords:** saccade control, lateral intraparietal area, countermanding task, motor inhibition, monkey

## Abstract

It is widely recognized that the posterior parietal cortex (PPC) plays a role in active exploration with eye movements, arm reaching, and hand grasping. Whether this role is causal in nature is largely unresolved. One region of the PPC appears dedicated to the control of saccadic eye movement—lateral intraparietal (LIP) area. This area LIP possesses direct projections to well-established oculomotor centers and contains neurons with movement-related activity. In this study, we tested whether these neurons are implicated in saccade initiation and production. The movement-related activity of LIP neurons was tested by recording these neurons while monkeys performed a countermanding task. We found that LIP neuronal activity is not different before the execution or the cancelation of commanded saccades and thereby is not sufficient for the initiation and production of saccades. Consistent with the evolutionarily late emergence of the PPC, this finding relegates the role of this PPC area to processes that can regulate but not trigger eye movements.

## 1 Introduction

Comparative studies have suggested that the posterior parietal cortex (PPC) arose from the concerted expansion of the somatosensory and visual cortices (Preuss, 2007; Ross and Martin, 2007), linking these sensory cortices to the motor areas of the frontal cortex (Kaas et al., 2011; Caminiti et al., 2015; Battaglia-Mayer and Caminiti, 2018; Kaas et al., 2018). This expansion parallels the growing importance of vision, hand dexterity, and eye-hand coordination in primates. These evolutionary considerations are commensurate with the widely recognized role of the PPC in active vision and touch. Based on these findings, we raise the question: must the PPC be therefore taken as exerting a causal role in the production of eye, hand, and arm movements?

Early neurophysiological experiments by Mountcastle et al. (1975) led to the influential proposal that PPC neurons function as a command apparatus for visual and tactile exploration of extra-personal space. Consistent with this proposal is the identification that, within the visual system, the PPC is part of the dorsal stream of information processing, which was considered initially to deal with spatial relationships—the “where” pathway (Ungerleider and Mishkin, 1982)—and subsequently to be more closely involved in movement planning and initiation—the “how” pathway (Goodale and Milner, 1992). Further support for this proposal is the body of evidence suggesting that PPC can be subdivided into regions specialized for different actions (Andersen and Buneo, 2002), such as reaching, grasping, and orienting gaze, as well as complex, coordinated movements that can be elicited by microstimulation (Cooke et al., 2003; Stepniewska et al., 2005). These specialized regions and their specific connections with frontal cortex motor regions thus form dedicated systems (Caminiti et al., 2017). Lastly, there is further evidence that PPC is functionally organized in its neurons displaying specific movement-related activity (e.g., Barash et al., 1991; Ferraina et al., 1997; Snyder et al., 1997; Gardner et al., 2007) as well as in the motor-related syndromes observed in parietal patients (Caminiti et al., 2010).

**Figure 1:**
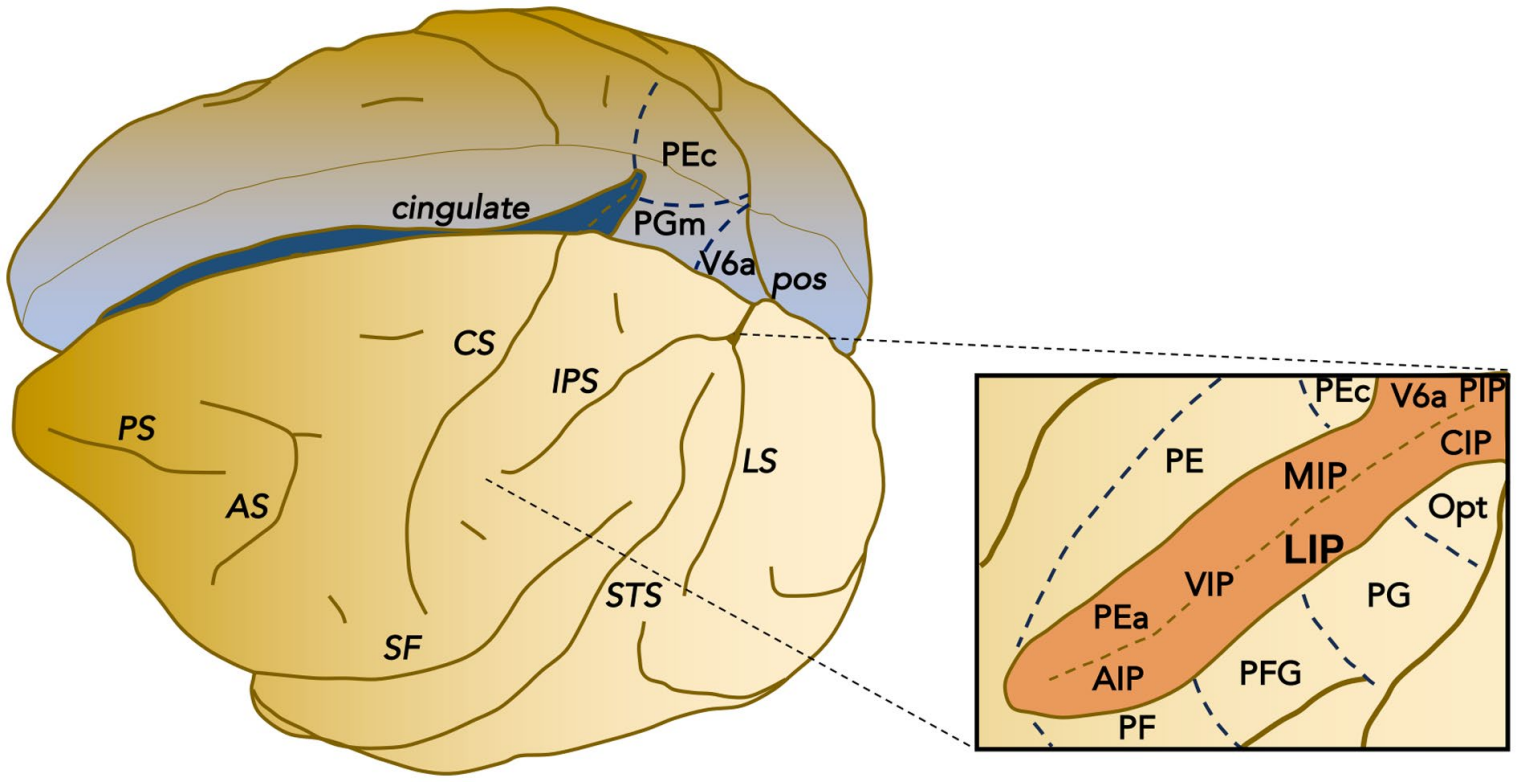
Parcellation of posterior parietal cortex of macaque. Schematic representation of the posterior parietal cortex areas within the intraparietal sulcus (IPS) and the surrounding surface. IPS delimits the superior and inferior parietal lobules. The posterior inferior parietal lobule includes area Opt, PG, and area LIP, CIP, and VIP within the lateral bank and fundus of the IPS. The posterior superior parietal lobule includes the IPS areas PIP and V6A, continuing on the mesial aspect with area PGm. The anterior inferior parietal lobule includes areas PFG and PF as well as area AIP within the IPS, while the anterior superior (medio-dorsal) parietal lobule includes PE and PEc (continuing on the mesial wall) as well as areas PEa and MIP within the medial bank of the IPS. Functionally, there is an anterior-posterior divide: areas within the anterior portion are primarily concerned with somatosensory processing, whereas areas within the posterior portion are primarily related to visual processing.

The command hypothesis recently received the strongest support in the finding that a neuronal population within the antero-dorsal aspect of the PPC—a subfield of areas PE and PEa (see Figure 1)—possesses projections that terminate within the spinal cord in the vicinity of last-order interneurons innervating distal hand motoneurons (Rathelot et al., 2017). This PPC region looks as an expansion of the parietal (area 2) corticospinal system with which it accounts for 17% of the disynaptic output from the cortex to spinal motoneurons (Strick et al., 2021). Its privileged access to the motor circuitry is further demonstrated by the observation that finger and wrist movements can readily be elicited by microstimulation delivered in that region (Rathelot et al., 2017). This finding strongly suggests that the PPC is causally implicated in active touch.

With respect to active vision and the voluntary control of saccadic eye movements, the evidence is not as compelling. One particular PPC subdivision—area LIP—has been attributed a role in saccade production, as evidenced by its distinctive movement-related activity (Andersen et al., 1990) and direct connections with established centers for oculomotor control: the midbrain superior colliculus (SC) (Paré and Wurtz, 1997) and the frontal eye field (FEF) within the prefrontal cortex (Ferraina et al., 2002). It has, however, not been demonstrated definitely that area LIP has a causal role in saccade production. Unlike the SC and FEF, area LIP does not project to the brainstem saccade burst generator.

One definitive test of whether a brain region possesses neuronal activity sufficient to account for saccade production is provided by the countermanding paradigm, which has been adapted for monkeys making saccadic eye movements (Hanes and Schall, 1996). This paradigm tests one’s ability to inhibit the initiation of a commanded response when an infrequent stop signal follows a go signal after a variable interval (stop-signal delay). Performance in this task can be modeled with a race between the go and stop processes leading up to either movement initiation or cancelation to estimate the length of time needed to cancel the commanded response (Logan and Cowan, 1984). Critically, neurons must meet two criteria to be involved in saccade production: (1) they must change their activity when a saccade is canceled instead of executed and (2) they must do so before that estimated time of saccade cancelation. This has been found to be the case for nearly all movement neurons tested in the SC (Paré and Hanes, 2003) as well as for nearly half of such neurons in the FEF (Hanes et al., 1998), thus establishing unequivocally the direct role of these brain regions in saccade production. In this study, we submitted LIP neuronal activity to this countermanding test.

## 2 Materials and methods

### 2.1 Subjects

Data were collected from four rhesus monkeys (*Macaca mulatta*, three females: 5.0–6.0 kg and one male: 10 kg) cared for and used under experimental protocols approved by the Queen’s University Animal Care Committee and in accordance with the Canadian Council on Animal Care guidelines.

Neuronal activity was recorded from the lateral bank of the intraparietal sulcus (IPS; Figure 1). The target area was accessed by a recording cylinder tilted 30° lateral or vertical, positioned over a hole trephined in the right hemisphere, and centered on stereotaxic coordinates P 5.0 and L 12.0 mm. The surgical procedures, stimulus presentation, and data acquisition have been described in detail previously (Shen and Paré, 2006). Both antibiotics and analgesic medications were delivered to the monkeys during the post-surgery recovery period. After recovery, the monkeys underwent operant conditioning and positive reinforcement procedures to perform fixation and saccade tasks for a liquid reward until satiation (Paré and Hanes, 2003; Thomas and Paré, 2007).

### 2.2 Behavioral tasks

**Figure 2:**
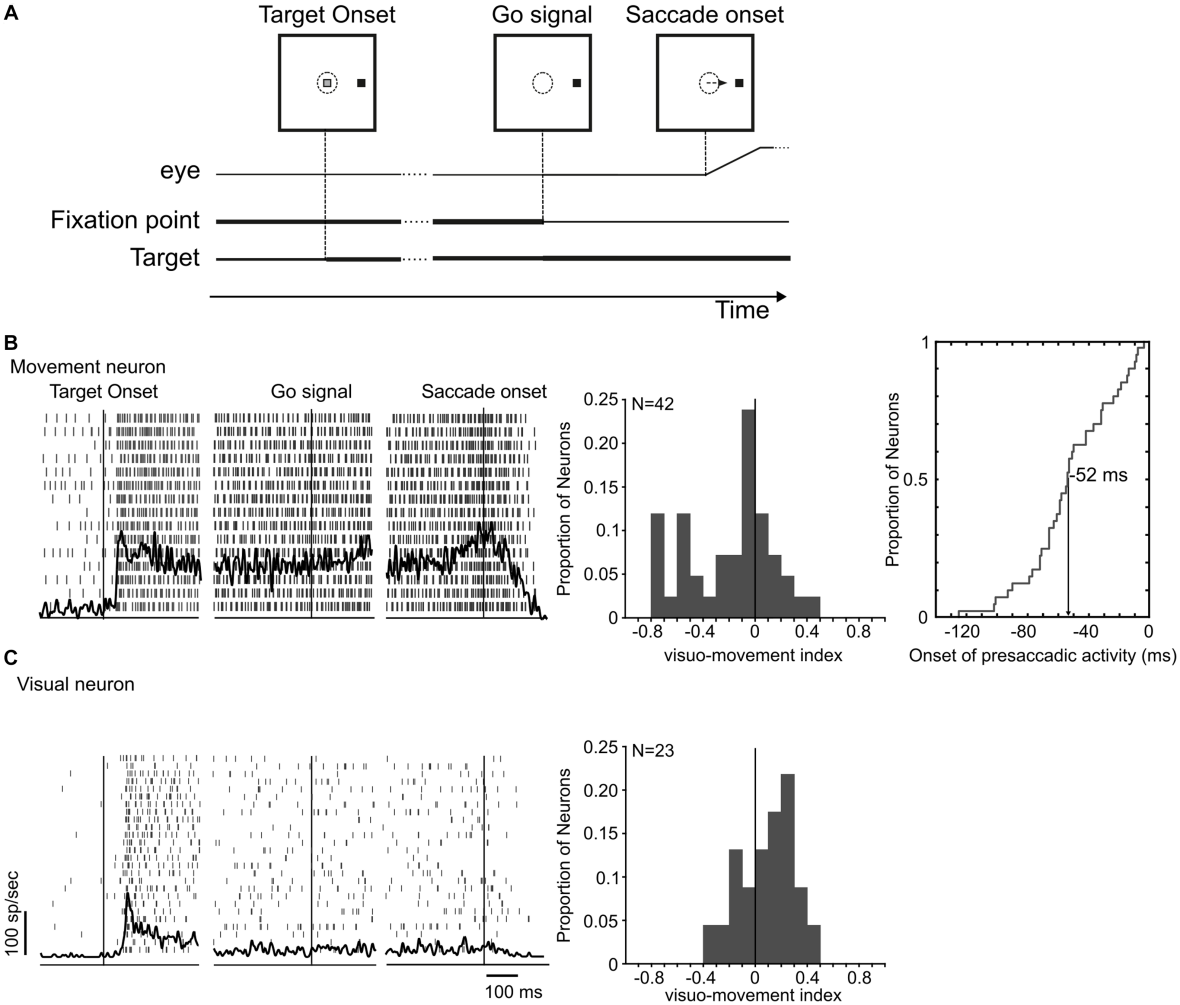
LIP neuronal responses in the visual delayed saccade task. **(A)** Monkeys were instructed to saccade to a peripheral target at the offset of the fixation point. **(B,C)** Neuronal profile of response of a movement and a visual neuron in area LIP during the preparation and execution of a saccade toward a target located at their response fields. Histograms in **(B,C)** display the distribution of visuo-movement index of both groups. For movement neurons, the distribution of the onset time of the saccade-related activity is plotted.

**Figure 3:**
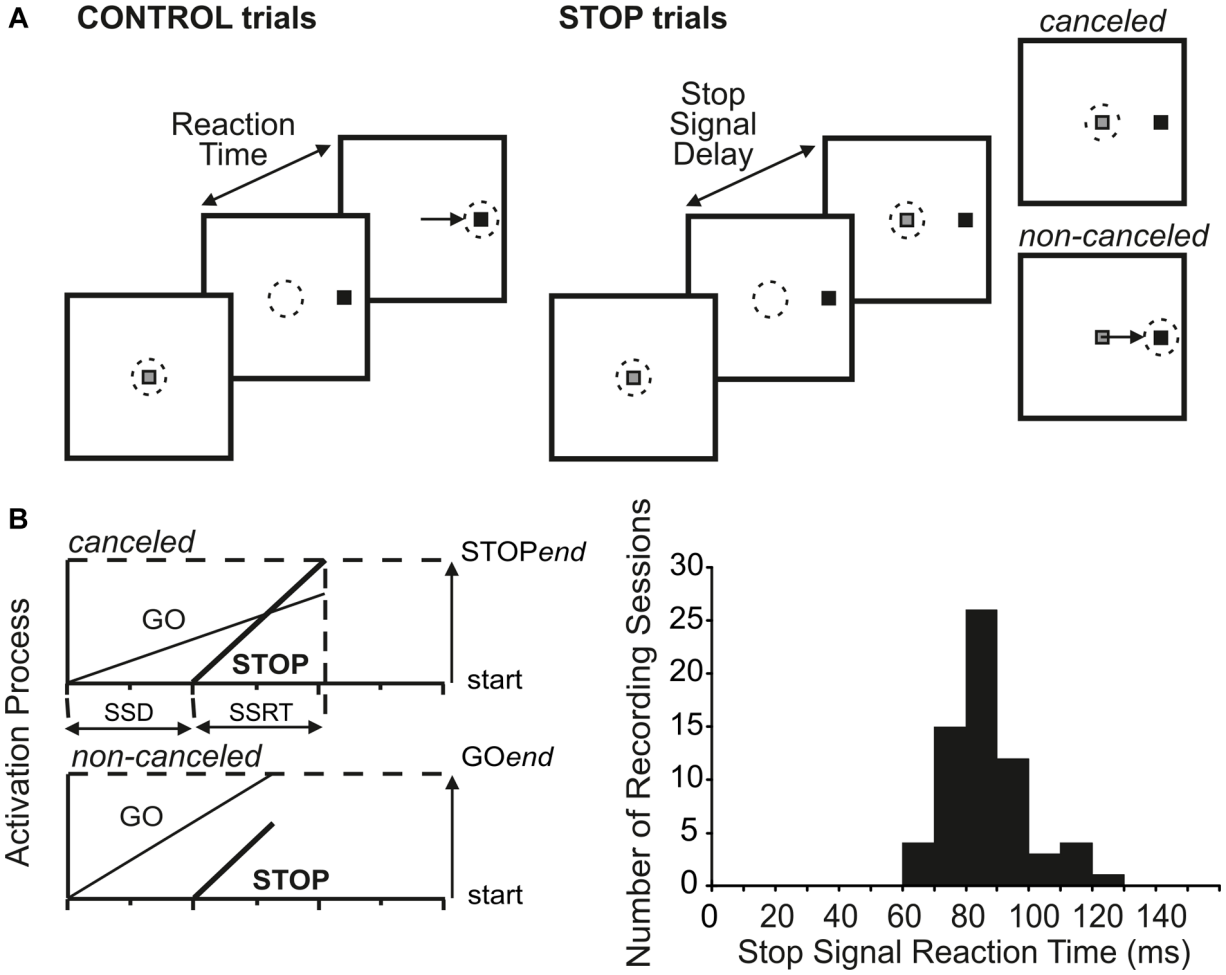
Countermanding paradigm and estimate of stop-signal response time. **(A)** In CONTROL trials, monkeys were reinforced for making a saccade to a visual go signal presented either in the center of the neuron’s response field or in the diametrically opposite direction. In STOP trials (33% of trials), the fixation spot reappeared after a variable delay following the go signal. Monkeys were reinforced if they withheld the commanded saccade (canceled STOP trials) but not if they failed (non-canceled STOP trials). **(B)** Race model and distribution of the estimated time needed for the stop process to be completed (saccade cancelation time), also known as the stop-signal response time (SSRT).

Four different eye movement tasks were presented to the monkeys for defining the properties of the studied neurons. The tasks included the visual delayed saccade task, the memory-guided saccade task, the gap saccade task, and the countermanding tasks (Paré and Wurtz, 2001; Paré and Hanes, 2003; Thomas and Paré, 2007). The main results reported here are obtained by the visual delayed saccade (Figure 2A) and the countermanding task (Figure 3A). The beginning of each task was signaled by the appearance of a central fixation spot, instructing the monkeys to catch it by its eye within 1,000 ms and keep fixating for a variable time of 500–800 ms. In the visual delayed saccade task, a peripheral target was presented, and the fixation spot remained illuminated for an additional 500–1,000 ms before disappearing to signal to the monkeys to make a saccade to the target within 500 ms and then maintain fixation on it for 200–400 ms. Trials that accomplished these requirements were rewarded. In the countermanding task (Figure 3A), after the initial fixation interval, a peripheral target appeared while the central fixation simultaneously disappeared. In these trials, the monkeys were required to make a saccade to the peripheral target within 500 ms and maintain fixation for the following 200–400 ms to obtain a liquid reward (CONTROL trials). In 33% of the trials (STOP trials), after a variable delay, referred to as the stop-signal delay (SSD), the fixation spot reappeared. To obtain the reward in these trials, the monkeys had to keep fixating the central spot for 600 ms (canceled trials). STOP trials where the monkeys broke the central fixation were not rewarded (non-canceled trials). Stop signals were presented at fixed SSD (four for each experimental session) ranging between 30 and 325 ms and spaced from 30 to 75 ms.

### 2.3 Data collection and neurons classification

Neuronal activity was recorded extracellularly, and the signal was sampled at 1 Hz to detect spike occurrence (Paré and Wurtz, 2001). Simultaneously, eye movements were tracked with either magnetic search coils (DNI, Newark, DE) or high-speed infrared cameras (Eyelink II, SR Research, Osgoode, Ontario, Canada) at a frequency rate of 1 kHz.

Within the PPC, area LIP (Figure 1) is most closely associated with eye movements (Andersen et al., 1990). This area can also be subdivided into dorsal (LIPd) and ventral (LIPv) portions based on cytoarchitecture and myelo-architecture, connections, and receptor distribution. LIPv contains a particularly dense layer III with large pyramidal neurons (Lewis and Van Essen, 2000b). LIPv is much more densely myelinated than LIPd (Blatt et al., 1990; Lewis and Van Essen, 2000b). LIPv is more connected with visual dorsal stream areas such as V3, MST, CIP, and V6, whereas LIPd is more connected with visual ventral stream areas such as V4 and temporal cortex areas (Lewis and Van Essen, 2000a; Galletti et al., 2001; Ungerleider et al., 2008; Mariani et al., 2019). LIPv is also more strongly interconnected with the FEF within the arcuate sulcus and sends more projections to the SC intermediate layers (Lynch et al., 1985; Andersen et al., 1990; Schall et al., 1995; Medalla and Barbas, 2006). In general, LIPv contains quantitatively lower neurotransmitter receptor densities than LIPd, especially lower NMDA, GABA_B_, GABA_A_/BZ, M1, alpha1, and 5-HT_1A_ receptor densities (Niu et al., 2020). Functionally, visual search accuracy has been reported to be selectively impaired by LIPv lesions (Liu et al., 2010); saccade reaction times are modestly increased by lesions of either subdivision (Li et al., 1999; Liu et al., 2010; Wilke et al., 2012). The great majority (88%) of the LIP neurons that we recorded were located at least 4 mm from the cortical surface and thus considered to be within area LIPv.

The discharge properties of LIP neurons were first characterized, while monkeys performed the visual delayed saccade task (Paré and Wurtz, 1997, 2001). Neurons were classified as movement neurons (Figure 2B) if they exhibited a significant increase in activity (>2 s.d. from the mean difference in delay period activity) preceding saccades. We classified those neurons that did not significantly increase their activity at the time around the saccade but exhibited a phasic response immediately after the target presentation in the response field as visual neurons (Figure 2C). Neurons classified as movement and visual were separately studied in the countermanding task. A visuo-movement index (VMI) that contrasts LIP visual and movement activity was calculated in the visual delayed saccade task as VMI = (visual − movement)/(visual + movement), where visual is the peak activity within 100 ms of visual stimulation and movement is the peak activity in the 40 ms preceding the saccade initiation (Thomas and Paré, 2007).

### 2.4 Data analysis

The beginning and termination of each saccade were computed off-line by setting a velocity and acceleration threshold as in the study by Waitzman et al. (1991). Saccade response times were computed as the time between the go signal and the saccade initiation. An experimenter verified the occurrence of the events detected by the algorithm. In STOP trials, we computed the probability to fail in canceling a saccade in function of the different SSD (inhibition function) and modeled the trend of this probability by a cumulative Weibull curve: *W*(*t*) = *γ* − (γ − δ) × exp.[−(*t*/*α*)*^β^*], where *t* is the time after target presentation, *α* is the time at which the inhibition function reaches 64% of its full growth, *β* is the slope, *γ* is the maximum value of the inhibition function, and *δ* is the minimum value of the inhibition function. All fitted functions of the different recording sessions had correlation coefficients >0.93 (mean 0.99) and amplitudes >0.55 (mean 0.79), with their upper and lower asymptotes approaching 1 (mean 0.85) and 0 (mean 0.07). Each function was used to estimate the SSD leading to fail the cancelation of half of the STOP trials. This value was used to obtain an average estimate of the time needed to cancel an instructed saccade (SSRT) by subtracting it from the average CONTROL response time (mean subtraction method). A second method (integration method) of estimate of the SSRT was implemented. By modeling each STOP trial as a race between a go and a stop process running independently toward a finish line, non-canceled trials can be approximated by the proportion of CONTROL trials sufficiently fast to escape the stop process for a given SSD. By integrating the distribution of the CONTROL response time, the upper limit of the response time of non-canceled trials, which approximates the end of the stop process, can be estimated. The SSRT is then obtained by subtracting the SSD from this value (Logan and Cowan, 1984; Paré and Hanes, 2003).

Neuronal activity was analyzed by computing spike density functions of each neuron’s discharge rates. For each trial, the time of each action potential was convoluted with a function modeling the rising and decay of postsynaptic potentials (Hanes and Schall, 1996; Paré and Hanes, 2003). The time course of the neuronal activation during STOP and CONTROL trials was compared by computing the point-by-point difference between the average spike densities of the two types of trials. The neuronal activity between CONTROL and STOP trials was taken as significant when the differential spike density function exceeded by 2 standard deviations (SD) ± the average difference in activity during the 200 ms preceding the go signal and remained above this threshold for 50 ms. We used the time at which this difference was stated to occur as a neuronal estimate saccade cancelation. Only data from STOP trials with stop-signal delays with at least eight trials were analyzed (with a mean of 23 and 19 for movement and visual-only neurons, respectively).

Our neuronal sample was further studied to evaluate a putative involvement of LIP in the proactive control of action. This control is expressed in the response time adjustment observed in CONTROL trials following a STOP trial: a longer response time than in CONTROL trials preceded by a CONTROL trial (Emeric et al., 2007; Pouget et al., 2011). Within the framework of the race model, this response time adjustment has been associated with a delayed onset of the ramping activity in FEF and SC neurons (Pouget et al., 2011). To search for a correlation of response time adjustment in LIP activity, in this study, we first selected the experimental session in which the behavioral adjustment was statistically significant, and then, we explored if that session influenced the way the neuronal activity evolved. In the present study, we focused our analysis on the neuronal baseline activity in the 100 ms preceding the go signal, the onset of ramping activity as in the study by Pouget et al. (2011), and the magnitude of visual response in the 25, 50, and 75 ms following the go signal.

Lastly, canceled and non-canceled STOP trials were studied to assess whether their neuronal correlate differed from the corresponding latency-matched CONTROL (longer than the SSD + SSRT for canceled STOP and shorter than SSD + SSRT for non-canceled STOP) trials. The difference in neuronal activity was taken as significant if the difference in the firing rate exceeded 2 SD ± the average difference in activity during the 200 ms preceding the target presentation and remained beyond this threshold value for at least 50 ms (Stuphorn et al., 2000; Ito et al., 2003).

## 3 Results

### 3.1 Neuron classification in the visual delayed saccade task

Our neuronal sample included 65 LIP neurons, which generally responded to the presentation of a visual stimulus in their receptive fields and continued to discharge until a saccade was made to that stimulus. Forty-two neurons (42/65, 65%) had a significant increase in activity before the visually guided response made in the delayed saccade task (Figure 2A) and were therefore identified as putative movement neurons. Figure 2B (leftmost panel) displays the activity of one neuron representative of this group. These neurons had, on average, a higher firing rate in the time preceding the saccade onset than after the target presentation (mean VMI: −0.20 ± 0.33) that started on average 52 ± 29 ms before the saccade onset (Figure 2B middle and rightmost panel). These neurons were analyzed separately from the remaining 23 neurons that showed only visually evoked responses and an average VMI of 0.12 ± 0.24 (Figure 2C).

### 3.2 Test of saccade control

The time course of the neuronal response of movement neurons was studied in the countermanding task (Figure 3A) to assess whether they carried a signal sufficient to control saccade’s production. The aim of the present analysis was to assess whether these movement neurons displayed a different profile of activation in the STOP trials with respect to the CONTROL trials and whether this difference was sufficiently early in time to control the saccade cancelation if it occurred. To obtain a behavioral estimate of saccade cancelation time, we fitted the race model to STOP trials as described in the Methods section (Figure 3B).

**Figure 4:**
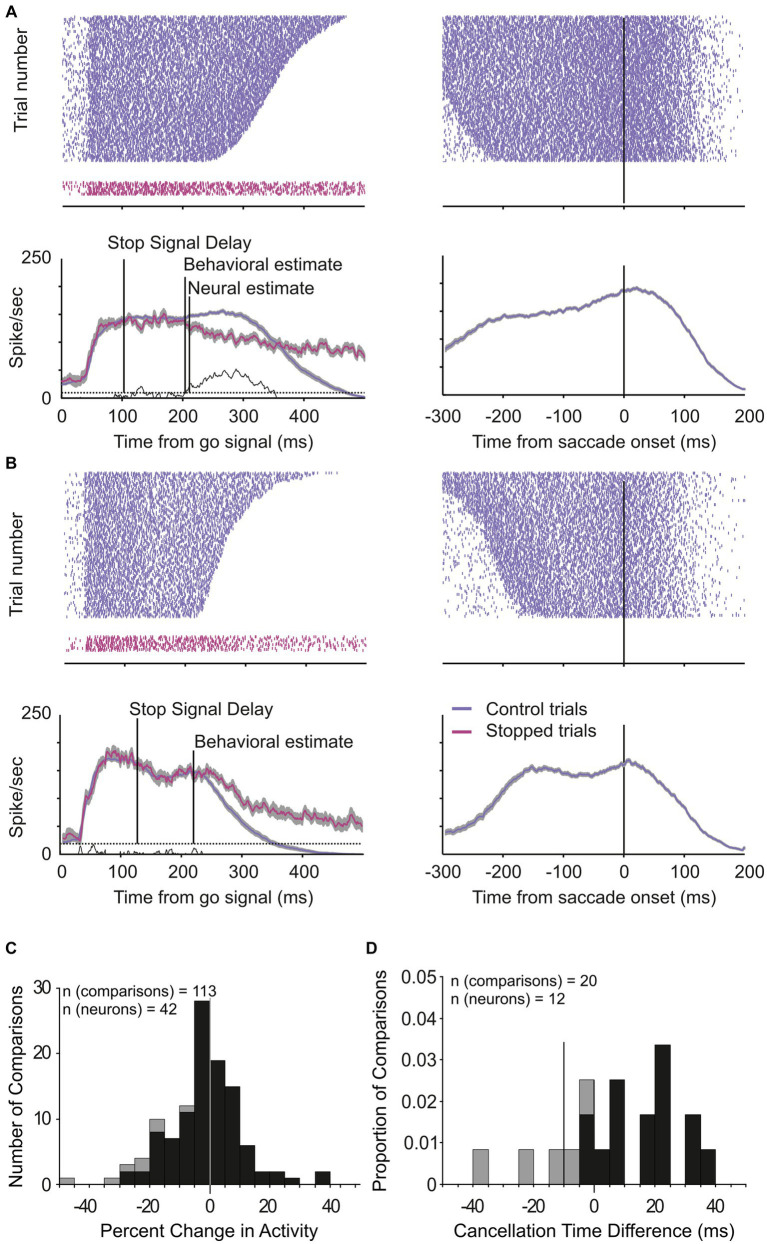
Change in LIP neuronal responses associated with saccade countermanding. **(A,B)** Activity of two LIP movement neurons in the countermanding task. Spike occurrences and average (±s.e.m.) activity in CONTROL (blue) and canceled STOP trials (magenta) aligned to the go signal (left) and saccades (right). Neuronal activity in CONTROL trials was taken from trials with response latency that exceeded the saccade cancelation time estimated from the race model, i.e., CONTROL trials in which a saccade would have been canceled if the stop signal had been presented. The ends of spike trains in left panels mark saccade onsets. **(C)** Distribution of percent change in activity for each LIP movement neuron (*n* = 42) at each possible stop-signal delay (113 comparisons). Negative percentages indicate less activity when a saccade is canceled than executed and positive more activity. **(D)** Distribution of the time difference between the neural and behavioral estimates of saccade cancelation (12 neurons, 20 comparisons). Vertical line at −10 ms marks the LIP efferent delay. Dark bars in both graphs indicate comparisons showing significantly lower activity in canceled STOP trials compared to CONTROL trials (7 neurons, 8 comparisons; *t*-test, *p* < 0.05).

Across the 65 experiments, we estimated that saccade cancelation took, on average, 87 ± 12 ms (Figure 3B). With this estimate in hand, we could then determine whether the LIP neurons recorded during the same sessions changed their activity to predict saccade production (Figures 4A,B). We first calculated the discharge rate of each neuron during a 40 ms interval centered on saccade cancelation time for both canceled STOP trials and corresponding CONTROL trials. Figure 4C shows the distribution of the percent change in activity calculated for each LIP movement neuron at each possible stop-signal delay. We found that the activity of these neurons was not significantly different when a saccade was canceled (mean −2.1 ± 13.0%; *t*-test: df = 112, *t* = −1.73, *p* = 0.09). Only 17% (7/42) of the neurons showed significantly lower activity following a stop signal presented in at least one stop-signal delay (*t*-test; *p* < 0.05). Evidently, the difference in activity of visual-only neurons was not statistically significant across the 65 possible comparisons (mean 5.6 ± 39.6%; *t*-test: df = 64, *t* = 1.15, *p* = 0.25). We next determined when LIP movement neurons changed their activity with respect to saccade cancelation time (Figure 4D). In only 12 neurons were we able to detect a change in neuronal activity in at least one stop-signal delay (20 out of 113 comparisons; Figure 4A, vs. Figure 4B), and these changes occurred before saccade cancelation only in a minority of the possible comparisons (7/20; 6/13 neurons). On average, the changes in LIP activity followed saccade cancelation by 7.6 ± 19.6 ms. Most importantly, only three LIP neurons (7%) significantly changed their activity within the minimal conduction time needed for LIP signals to reach the eye muscles—this efferent delay is estimated to be the same as for FEF, i.e.,10 ms (Paré and Hanes, 2003). Overall, 93% (39/42) of LIP movement neurons did not change their activity to predict saccade production.

The very few neurons that changed their activity when a saccade was canceled tended to have greater movement than visual activity (visuo-movement index: −0.35 vs. −0.14; *t*-test: df = 40, *t* = 1.97, *p* = 0.06) and earlier pre-saccade increase in activity (−63 vs. −47 ms; *t*-test: df = 40, *t* = 1.65, *p* = 0.11), as determined in the visual delayed saccade task. We could not determine a change in activity from any of the visual-only neurons.

**Figure 5:**
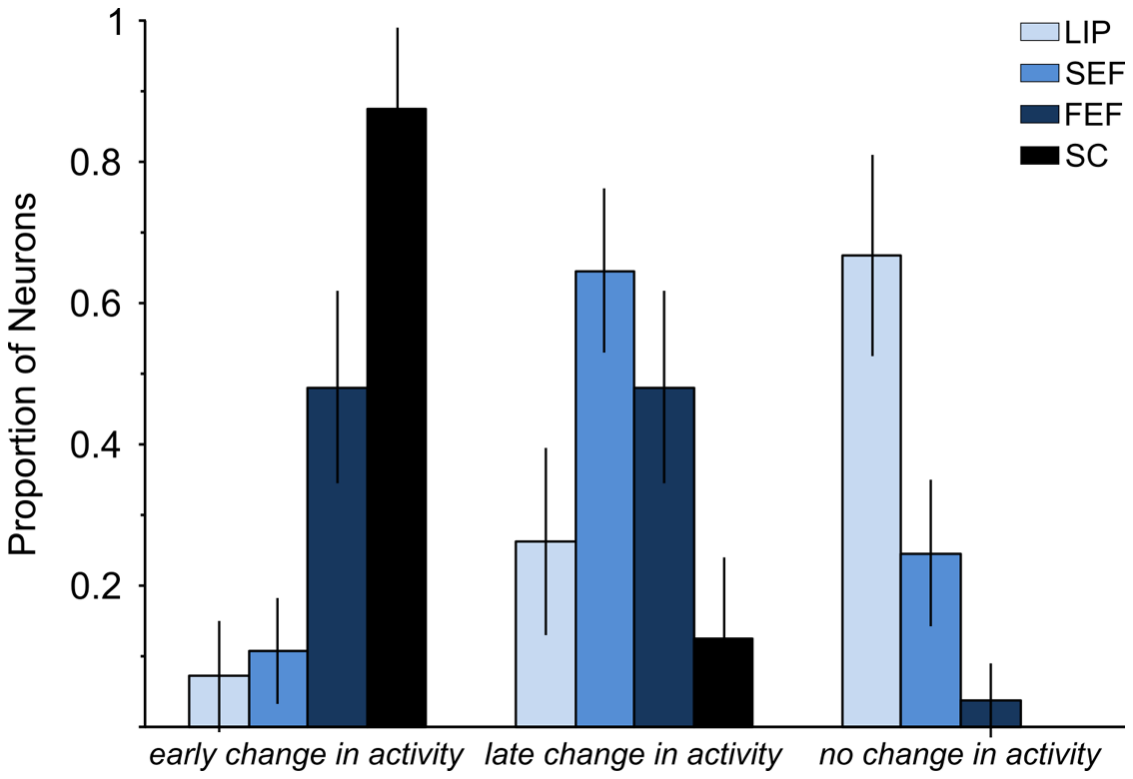
Comparison of LIP, FEF, and SC neuronal responses associated with saccade countermanding. Graph shows the percentage of neurons showing (1) a significant change in activity occurring early enough to account for saccade cancelation, i.e., within the efferent delay (*early change in activity*); (2) a significant change in activity but occurring after saccade cancelation (*late change in activity*); and (3) no significant change in activity around the time of saccade cancelation (*no change in activity*). Data are from samples of neurons: 42 in LIP, 65 in SEF (Stuphorn et al., 2010), 51 in FEF (Hanes et al., 1998), and 32 in SC (Paré and Hanes, 2003). The efferent delay is the minimal conduction time needed for signals from LIP, SEF, FEF, and SC to reach the eye muscles: 10 ms for LIP, SEF, and FEF and 8 ms for SC (Paré and Hanes, 2003).

Figure 5 shows the proportion of neurons modulated in each of the brain regions that have been investigated so far. Statistically, the small proportion of LIP neurons significantly modulated before countermanded saccades (3/42 = 0.071 ± 0.078 95% C.I.) is not statistically different from 0 (Fisher’s exact test, *p* = 0.24). It is significantly lower than that observed in SC (Paré and Hanes, 2003) and FEF (Hanes and Schall, 1996) (28/32 and 25/52, Fisher’s exact test, *p* < 0.0001) but not statistically different from that reported in the supplementary eye field (SEF) study of Stuphorn et al. (2010) (3/42 vs. 7/65; Fisher’s exact test, *p* = 0.74). It is worth mentioning that the latter study came to the same conclusion as we have for LIP, that is, SEF does not have a direct control in saccade initiation.

**Figure 6:**
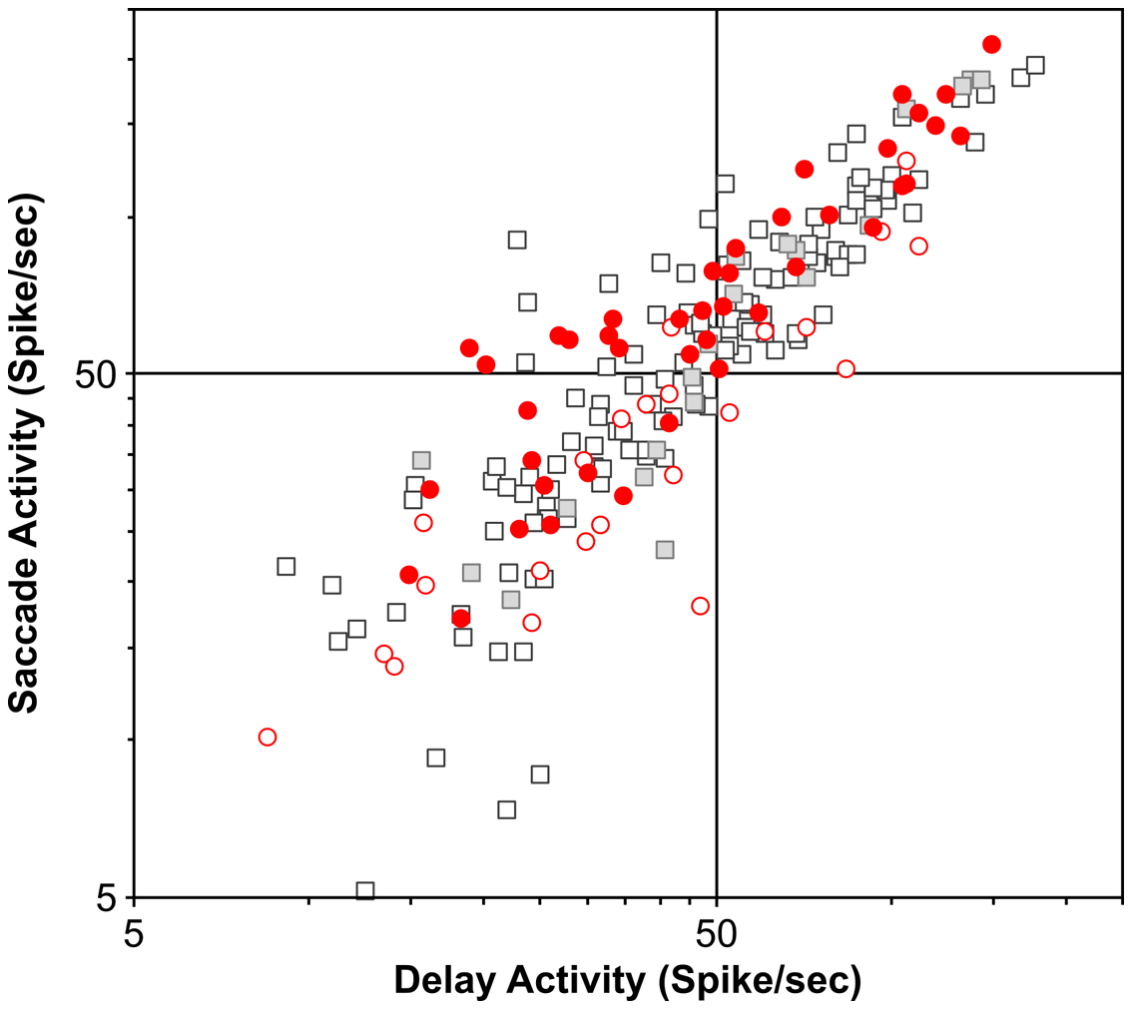
Functional properties of the present population and previously recorded populations of LIP neurons in the visual delayed saccade task. Scatter plot of the delay period and saccade-related activity does not reveal any clustering of the different populations of LIP neurons. Furthermore, it shows that this study’s neuronal sample does not possess specific properties. Circles, visual neurons in this study (*n* = 23); red circles, movement neurons in this study (*n* = 42); squares, additional neurons recorded in the laboratory (*n* = 128); and gray squares, neurons antidromically activated from SC stimulation (*n* = 21).

In our study, we carefully selected the most likely candidate neurons to test our hypothesis, and our sample size compares well with other samples, including that in the FEF (*n* = 51) and SC (*n* = 32) studies as well as in LIP studies of projection neurons. Nevertheless, the possibility arises that perhaps the few putative movement neurons that were modulated before countermanded saccades are projection neurons with terminals in the SC. We compared the salient discharge properties of our neuronal sample as assessed in the visual delayed saccade task with those of the neurons recorded in the same task in our laboratory. Figure 6 shows the plots of pre-saccade activity against delay period activity for all 65 (movement and visual) neurons; pre-saccade and delay activity of this sample averaged 65 ± 6 sp/s and 53 ± 4 sp/s, respectively. This plot also contrasts this activity relationship with a separate sample of 149 neurons recorded in our laboratory, including 21 neurons antidromically activated from the SC; pre-saccade and delay activity of this sample averaged 61 ± 4 sp/s and 50 ± 3 sp/s, respectively. As can be seen, the sample of neurons recorded in our countermanding study is representative of the LIP population. There is no statistically significant difference between these samples (*t*-test, *p* = 0.70 and 0.84 for pre-saccade and delay activity, respectively). Our study sample was also not statistically different from the 40 LIP neurons antidromically activated from the SC by Paré and Wurtz (2001) (*t*-test, *p* = 0.60 and 0.55 for pre-saccade and delay activity, respectively). We suppose that these comparisons strengthen the evidence put forth in our study.

Consistent with the results described above, a separate analysis of the STOP trials revealed that the activity of LIP movement neurons does not predict the behavioral outcome of these trials in contrast to what has been found for FEF and SC neurons (Lo et al., 2009). This analysis compared early activity in canceled and non-canceled STOP trials during a 50 ms interval ending with either the go or the stop signal. We did not find elevated LIP activity in advance of saccades by the time that the go signal was presented (23 vs. 28 sp/s; paired *t*-test: df = 32, *t* = −1.43, *p* = 0.16) or when the stop signal was presented (67 vs. 70 sp/s; paired *t*-test: df = 32, *t* = −0.74, *p* = 0.46).

**Figure 7:**
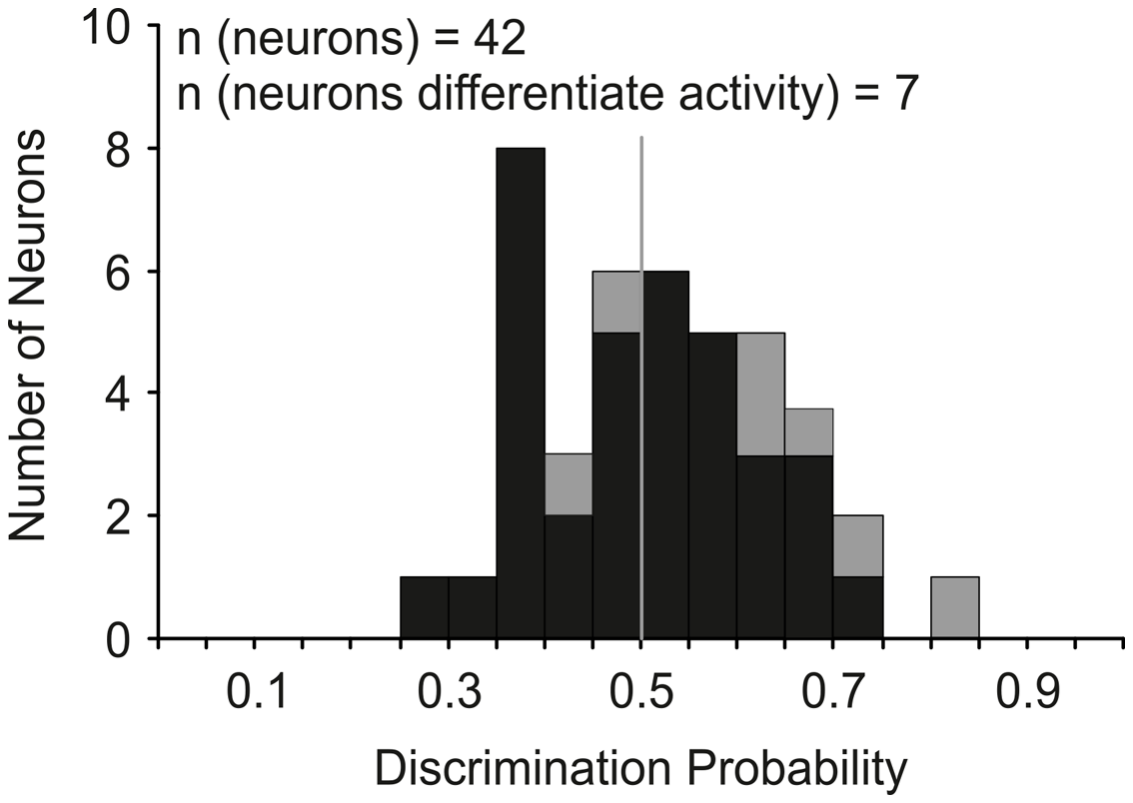
Discrimination of LIP neuronal responses associated with saccade production and countermanding. Distribution of the probability of an ideal observer’s discriminating the activity of LIP movement neurons (*n* = 42); probability of 1 indicates LIP saccade-related activity both greater and distinct from cancelation-related activity and probability of 0 indicates the converse. Gray bars indicate the neurons (*n* = 7) that showed significantly lower activity in canceled STOP trials compared to CONTROL trials (*t*-test, *p* < 0.05) in at least one stop-signal delay.

Our next analysis tested the hypothesis that the activity of LIP movement neurons must surpass a trigger threshold for a saccade to be produced as has been shown for FEF (Hanes and Schall, 1996) and SC (Paré and Hanes, 2003) neurons. We used an ideal observer discriminant analysis (Brown et al., 2008) to compare the maximum discharge rate of each neuron during the last 20 ms before saccade initiation in CONTROL trials with the maximum rate observed during the 40 ms interval centered on saccade cancelation time in the canceled STOP trials. Figure 7 shows how the distribution of discrimination probability across LIP movement neurons was not significantly different from chance (mean 0.52 ± 0.12; *t*-test: df = 41, *t* = 0.94, *p* = 0.35).

### 3.3 Test of adaptive response time adjustment

**Figure 8:**
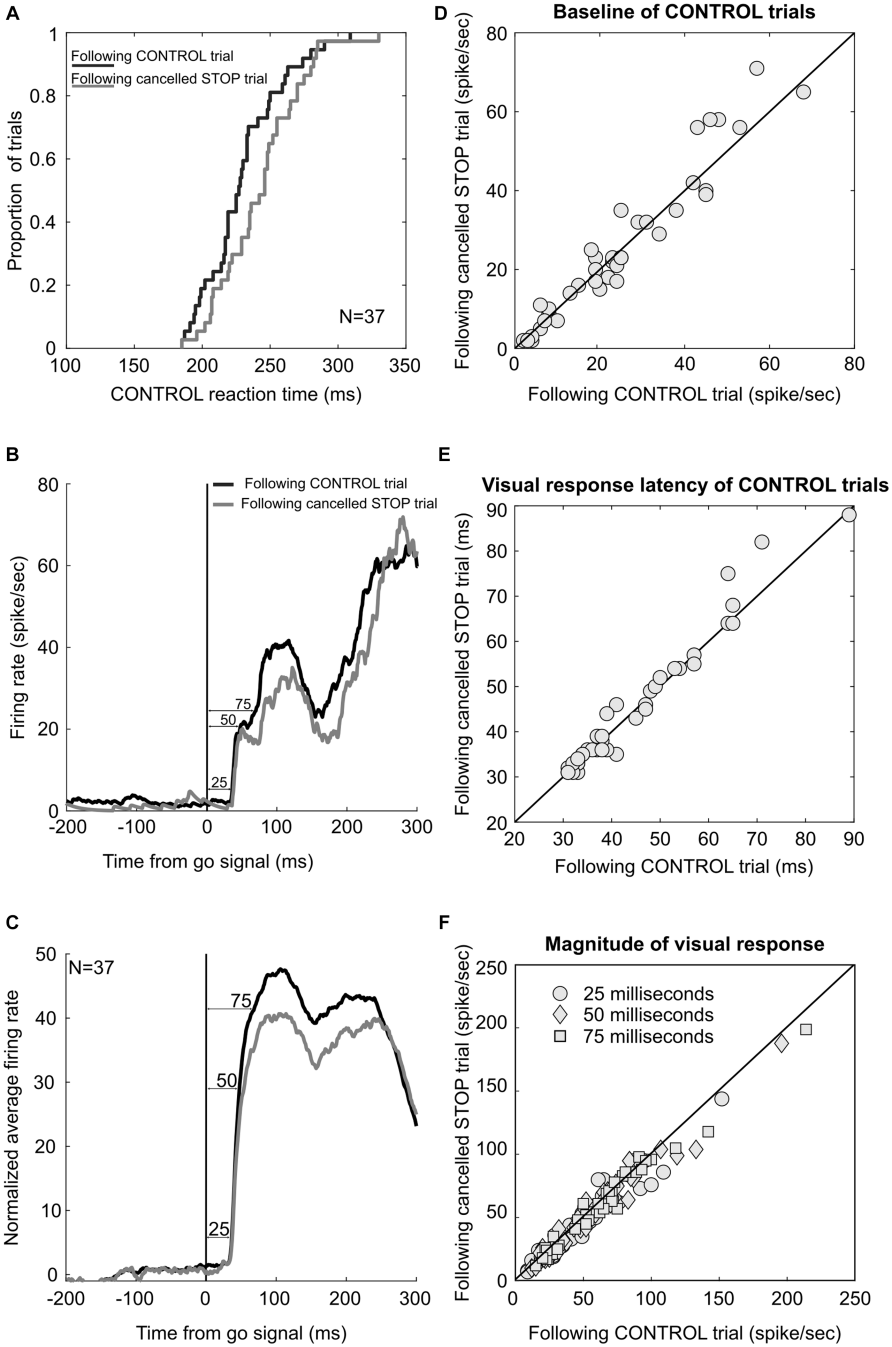
LIP neuronal correlates of post-STOP trial adjustment. **(A)** Cumulate distribution of response time in CONTROL trials following correct canceled STOP trials (gray) and CONTROL trials (black). **(B)** Neuronal response of an example LIP neuron in CONTROL trials following correct canceled STOP trials (gray) and CONTROL trials (black). **(C)** Average activity across the sample of 37 LIP neurons. Neuronal activity was obtained as the difference of each spike density function value from the average activity in the 200 ms preceding the go signal. **(D)** Scatter plot showing baseline activity in CONTROL trials following correct canceled STOP trials and CONTROL trials. **(E)** Scatter plot showing the latency of visual responses in CONTROL trials following correct canceled STOP trials and CONTROL trials. **(F)** Scatter plot showing significantly lower activity in CONTROL trials following correct canceled STOP trials than those following CONTROL trials in the 25, 50, and 75 ms following the target onset.

Previous studies have shown that macaque monkeys performing a saccade countermanding task like humans adjust their response time adaptively, responding slower after STOP trials than after CONTROL trials (Emeric et al., 2007; Pouget et al., 2011). This response time lengthening after successfully canceled STOP trials is accompanied by a delay in the onset of the activation of FEF and SC movement neurons, rather than by a change in the threshold, baseline, or accumulation rate (Pouget et al., 2011). In contrast, the activation of visually responsive FEF and SC neurons is not delayed, thus refuting the hypothesis that the RT lengthening in this task was due to an adaptive adjustment in visual processing preceding the activation of movement neurons. Here, we further tested this hypothesis and the contribution of LIP neurons in this adaptive response time adjustment, which our monkeys also displayed. Across the 65 sessions in which LIP neurons were recorded, response time in CONTROL trials preceded by a canceled STOP trial was significantly longer than response time in CONTROL trials preceded by a CONTROL trial (258 vs. 250 ms, paired *t*-test *p* < 0.001). This response time adjustment was significant in two of our monkeys, together showing an average slowing rate of 14 ms (244 vs. 230 ms; *p* < 0.001) across 37 sessions, in which 25 movement and 12 visual-only neurons were recorded. All these neurons displayed visually evoked responses within 100 ms of the visual stimulation, but we found no difference in the onset of these responses measured in the different trial sequences (46 vs. 45 ms; paired *t*-test, *p* = 0.25), thus adding further support for the hypothesis that there is no adaptive adjustment in visual processing (Figure 8).

However, the magnitude of these responses was found to be significantly less in CONTROL trials preceded by a canceled STOP trial, as measured as the mean spike density function during the first 25 ms of the response (52 vs. 55 sp/s; paired *t*-test, *p* = 0.03). This was also the case when we measured the activity during the first 50 ms (60 vs. 64 sp/s; *p* = 0.01) or 75 ms (62 vs. 66 sp/s; *p* = 0.002). We observed a difference neither in the baseline activity measured during the last 100 ms before the target onset of these 37 neurons (25 vs. 24 sp/s; *p* = 0.26) nor in the threshold level of the 25 movement neurons (70 vs. 71 sp/s; *p* = 0.65). In summary, RT lengthening following canceled STOP trials was not associated with delayed activation of LIP neurons, but the magnitude of that activation was significantly attenuated. This latter result contrasts with those from FEF and SC neurons, even though the RT lengthening was comparable. Nevertheless, the effect size was small, and we noted that less than half of the neuronal sample showed significant modulation: 30%, 43%, and 46% in the 25, 50, and 75 ms epoch, respectively.

### 3.4 Test of performance monitoring

**Figure 9:**
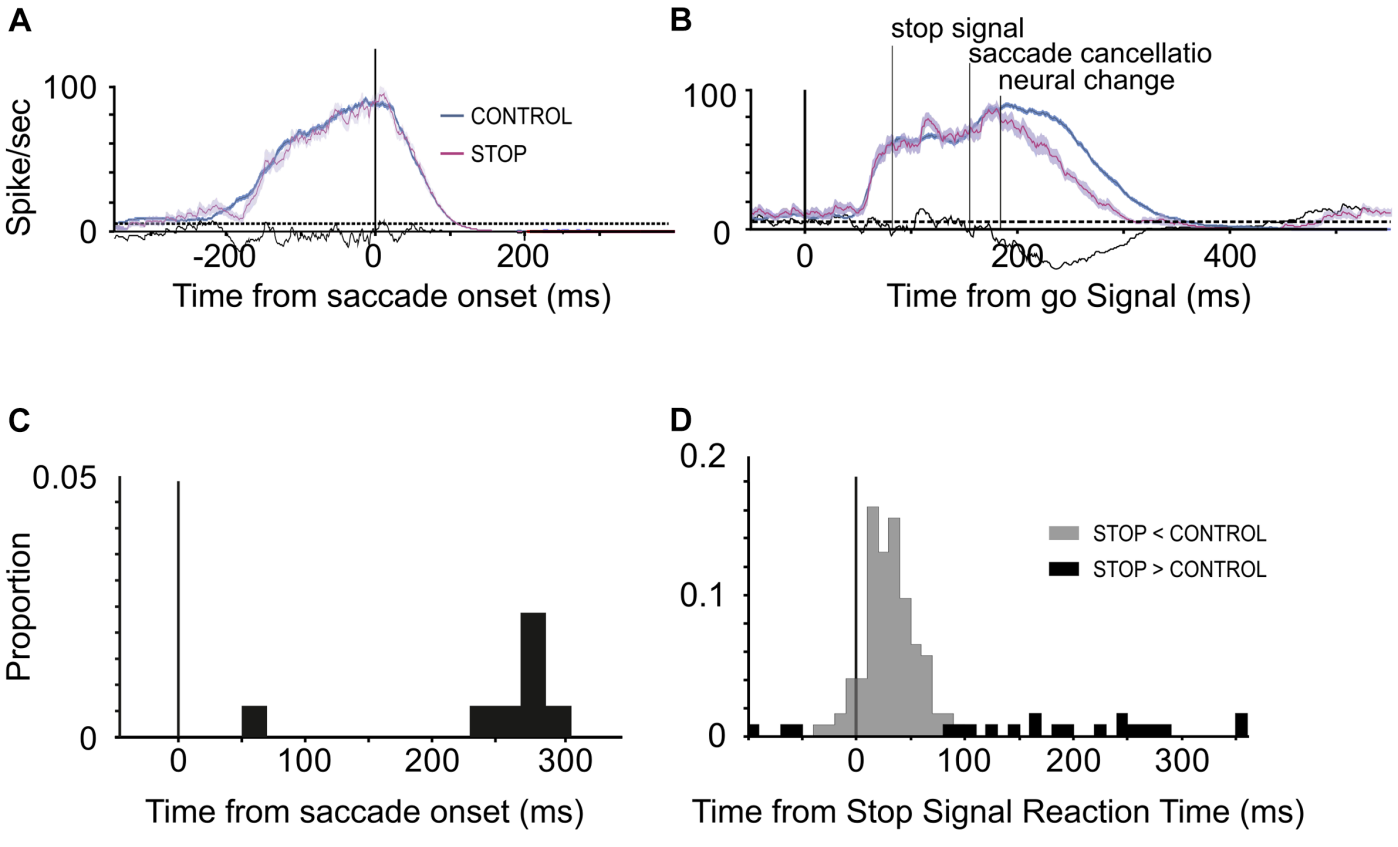
LIP neuronal responses associated with error and conflict detection. Average (±s.e.m.) activity in non-canceled STOP trials (**A**; magenta) and canceled STOP trials (**B**; magenta) contrasted to the corresponding latency-matched CONTROL trials (**A,B**; blue) aligned on saccade onset. Distribution of the time differences of non-canceled **(C)** and canceled **(D)** STOP trials with the latency-matched CONTROL trials. Observations in which the discharge rate of STOP trials was higher (black) or lower (gray) than CONTROL trials are indicated.

Finally, we assessed the possible role of LIP neurons in either detecting the subject’s errors or signaling instructions’ conflict monitoring (Stuphorn et al., 2000; Ito et al., 2003). Specifically, we tested (1) whether LIP neuronal activity immediately following erroneously performed saccades in STOP trials was significantly higher than in latency-matched CONTROL trials (Figure 9A) and (2) whether LIP neuronal activity during the canceled STOP trials was significantly higher than the latency-matched CONTROL trials following the appearance of stop signal (Figure 9B). When examining error detection, only four of the 42 movement neurons increased significantly their activity in error STOP trials with respect to the latency-matched CONTROL trials for at least one stop-signal delay (Figure 9C). Similarly, very few neurons (8/42) were signaling a conflict between instructions by a change in activity in STOP trials with respect to the latency-matched CONTROL trials (Figure 9D, black bars). On the contrary, the great majority of the movement neurons (33/42) decreased their activity approximately 25 ms after the stop-signal response time (Figure 9D, gray bars). In summary, the activity of our LIP neuronal sample reflected neither a putative error signaling nor a putative task conflict monitoring signal.

## 4 Discussion

Our study shows that LIP neuronal activity does not meet the criteria to be sufficient for saccade production. The best candidate neurons, those with movement-related activity, were found to change their activity neither significantly nor early enough when a saccade is executed instead of canceled, thus demonstrating that they cannot generate motor commands. These results stand in stark contrast to those obtained from FEF and SC countermanding studies (Figure 5) and thus resolve what has been an enduring issue.

Our results, at least in part, are not dissimilar to those obtained with the testing of SEF neurons in the countermanding paradigm. Despite the presence of pre-saccade activity observed in this prefrontal cortex area since its identification, neurons with such activity failed to change their activity early enough when a saccade was executed instead of canceled (Stuphorn et al., 2010). However, while the properties of SEF neurons in detecting canceled and non-canceled saccades provide to this brain area a role in the proactive control of eye movements (Ito et al., 2003), the same role cannot be attributed to LIP. The same conclusion is prompted by the observation of a lack of significant adjustment in the baseline activity, the onset time of activation, or the ramping of activity associated with the slowing of response time in CONTROL trials following canceled STOP trials, a result contrasting the observation in FEF and SC (Pouget et al., 2011). On the contrary, consistent with a proactive control of saccade, here we observed a proportion of LIP neurons showing a significantly lower activity in the CONTROL trials following canceled STOP trials. This result is in line with the role of LIP in encoding a variety of contextual information, including internal variables, for engaging in the proper behavioral response (Huk et al., 2017).

The neuronal activity observed in LIP during the executed and canceled movement is rather consistent with the detection of eye position and therefore with the encoding of an efferent copy of the motor command for controlling the current saccade, possibly received from FEF or SC by their interconnections (Paré and Wurtz, 1997; Ferraina et al., 2002). The capability of LIP of remapping the position of salient stimuli in the eye position reference frame (Duhamel et al., 1992) suggests that this brain area could be directly involved in encoding corollary discharge signals for eye movements (Hall and Colby, 2011). Further studies, purposely designed, will be needed to address whether LIP, as the SC, the FEF, and the MD thalamus (Crapse and Sommer, 2008), is a node of the pathway carrying the corollary discharge signals subtending the stability of the visual scene (Wurtz, 2008; Cavanaugh et al., 2016).

Together, our findings in LIP and those in SEF indicate that the mere existence of a pre-saccade increase in the activity of a neuron cannot be taken as evidence that this neuron is involved in saccade production. For example, some neurons in the visual cortex have been shown to increase their activity just prior to a saccade made to a stimulus presented in their receptive fields (Moore, 1999; Nakamura and Colby, 2000; Supèr et al., 2004), but no one would readily assign a role in saccade initiation to this seemingly “movement-related” activity. A recent study indicates that such activity in visual cortex neurons reflects pre-saccadic attention (Hanning et al., 2023). We, therefore, predict that any “movement-related” activity in visual cortical areas would not pass the countermanding test, and we suggest that LIP “movement-related” activity also reflects pre-saccadic attention.

The failure of LIP neuronal activity to pass the countermanding test can be reconciled with several previously reported observations. First, saccade production is not impaired following reversible inactivation of area LIP (Li et al., 1999; Wardak et al., 2002; Liu et al., 2010; Wilke et al., 2012). In fact, only a moderate increase in saccade latency is typically noted. Similar observations were made following ablation, but, in those cases, the monkeys suffered from visual extinction^1^ (Lynch and McLaren, 1989): They tend to ignore a contralateral stimulus when presented together with an ipsilateral one. A saccade bias toward the ipsilateral stimulus is also often observed following reversible inactivation in the same experimental condition (Schiller and Tehovnic, 2003); conversely, microstimulation induces a saccade bias toward the contralateral stimulus (Schiller and Tehovnik, 2001). A likely explanation for these observations is that LIP neuronal activity contributes to the process of saccade preparation, even though it does not contribute to the saccade trigger. Thus, a diminished preparation delays the initiation of the saccades (see Paré and Munoz, 1996). In fact, any reduction in the inputs to the saccadic eye movement system may prolong saccade latency (see Zhang and Fries, 2023). This may explain the particularly long latencies of visually guided saccades following combined FEF and LIP lesions (Lynch, 1992). As evidenced by normal spontaneous saccades, saccade production was nonetheless preserved in those cases, unlike when both FEF and SC were lesioned (Schiller et al., 1980). Interestingly, patients with a lesion of the parieto-collicular tract show normal saccade latency (Gaymard et al., 2003). The deficit of these patients is limited to their contralateral saccades being hypometric in a spatially unpredictable saccade task but normal in predictable and memory-guided saccade tasks.

Second, area LIP microstimulation does elicit saccades but rather only when a large electrical current is delivered (Shibutani et al., 1984; Kurylo and Skavenski, 1991; Thier and Andersen, 1998; Mushiake et al., 1999). The direct projections of LIP neurons to the SC intermediate layers or the FEF certainly underlie this effect. It is noteworthy that Ferrier (1875) and later Vogt and Vogt (1926) reported that eye movements were much more difficult to evoke from stimulating field 7a than occipital area 19a (area V4). Similarly, Foerster (1931) rarely could evoke eye movement in humans by stimulating the parietal adversive field, even with a strong current.

Third, LIP movement-related activity changes with visual context: it is generally reduced when saccades are not visually guided (Andersen et al., 1987; Paré and Wurtz, 1997, 2001; Gottlieb and Goldberg, 1999; Ferraina et al., 2002) as well as when several stimuli are present (Thomas and Paré, 2007). It cannot therefore reflect a saccade trigger signal. Nevertheless, it must be noted that the absence of evidence in our study for a saccade trigger threshold in the activity of LIP neurons is not at odds with the observation that LIP activity, when recorded in a visual motion discrimination task with varying levels of difficulty, converges to a single level about 60 ms before saccade initiation (Roitman and Shadlen, 2002; Churchland et al., 2008). This activity coalescence has been interpreted as a decision threshold, but its timing clearly makes it distinct from the saccade trigger threshold, which ought to be reached approximately 10–12 ms before saccade initiation (Paré and Hanes, 2003) to be consistent with the anatomy and physiology of the pre-motor circuitry of the saccadic eye movement system (Hanes and Schall, 1996). This early activity threshold therefore cannot be taken as evidence that LIP neuronal activity regulates saccade production. The evidence of a lack of a direct control of LIP on saccade production become of relevance for adjusting the weight of eye movement-related signals carried by LIP perisaccadic activity during perceptual decision (Park et al., 2014) and shed light on the contribution of this area on cognitive functions (see Huk et al., 2017 for review).

Human imaging studies have identified a response inhibition cortical network, which includes the right inferior frontal gyrus (IFG), the pre-supplementary motor area (preSMA), and the IPS (Rae et al., 2014; Hampshire and Sharp, 2015; see also Curtis et al., 2005; Xu et al., 2017; Jarvstad and Gilchrist, 2019). Of these regions, only activation in the right IFG is correlated with SSRT. Recently, Osada et al. (2019) delivered transcranial magnetic stimulation (TMS) pulses over an IPS region functionally connected with the right IFG and preSMA, while participants performed a visuo-manual countermanding task involving a button press. TMS disrupted cancelation: It prolonged the SSRT. This effect was, however, restricted to a very late period into the task, when stimulation was delivered in the last 30 ms before canceling. These results are difficult to interpret. First, it is difficult to rule out completely that the stimulation could have exerted its effects by indirectly activating the connected frontal areas. Second, there is some indication that TMS marginally increased GO response time in that time period; however, this outcome should have facilitated, not impaired, cancelation [i.e., shorten and not prolong SSRT; see (Stuphorn and Schall, 2006)]. A likely explanation is that TMS selectively disrupted inhibitory elements, perhaps exclusively involved in hand movement control (Desmurget et al., 2018). Equally possible is that the TMS pulses in the study by Osada et al. (2019) activated the visual fixation neurons that are common in PPC (Mountcastle et al., 1975; Lynch et al., 1977), which may account for the PPC activation in inhibitory control tasks; it is noteworthy that LIP microstimulation elicits saccades followed by suppression (Constantin et al., 2009). We did not record this type of neuron in our study, which aims to test the role of area LIP in saccade initiation, not inhibition.

Our findings are also consistent with the view that the role of PPC in regulating saccadic eye movements is evolutionarily recent (Paré and Dorris, 2011). Together with the prefrontal and the superior temporal cortices, the PPC is a node within a “higher-order” network, which is unique to old-world monkeys and apes (Preuss, 2007). In comparison, the FEF is conserved across primates (Preuss, 2007; Kaas, 2008), and the SC (optic tectum) is conserved across vertebrates (Butler and Hodos, 2005). Furthermore, the PPC tectal projections are negligible in new-world monkeys (Collins et al., 2005). Overall, primates evolved from ancestors that possessed a very small portion of their cerebral cortex that can be identified as PPC (Kaas et al., 2011). The evolutionarily late emergence of PPC in the eye movement circuit appears to be concomitant with an oculomotor range expansion, which demands enhanced control over visual information processing, such as target selection, that active vision requires. Consistent with the principle that the basic organization of neural circuits is evolutionarily conservative (Katz and Harris-Warrick, 1999), the expansion of the PPC could have offered behavioral changes through adaptive changes in modulatory inputs to the oculomotor control network. Area LIP would thus only be indirectly involved in saccade control, and it could serve primarily to enhance the sensory guidance and flexible regulation of saccades. In line with this idea is the evidence that area LIP integrates sensory and goal-directed information into a salience map wherein objects are selected for further processing, such as to direct gaze (Thomas and Paré, 2007; Bisley and Goldberg, 2010). Such a map is integral to models of visual search and attention (Tsotsos, 2011).

## Data availability statement

The raw data supporting the conclusions of this article will be made available by the authors, without undue reservation.

## Ethics statement

The animal study was approved by Queen’s University Animal Care Committee. The study was conducted in accordance with the local legislation and institutional requirements.

## Author contributions

EB and MP contributed to the collection and analysis of the data and wrote the manuscript. MP conceived the study. All authors contributed to the article and approved the submitted version.

## Footnotes

^1^Lesions of the human inferior parietal lobule induce neglect, but only lesions involving the superior temporal sulcus (STS) have been reported to induce neglect in monkeys (Watson et al., 1994).

## References

[ref1] Andersen R. A. Asanuma C. Essick G. Siegel R. M. (1990). Corticocortical connections of anatomically and physiologically defined subdivisions within the inferior parietal lobule. J. Comp. Neurol. 296, 65–113. doi: 10.1002/cne.902960106, PMID: 2358530

[ref2] Andersen R. A. Buneo C. A. (2002). Intentional maps in posterior parietal cortex. Annu. Rev. Neurosci. 25, 189–220. doi: 10.1146/annurev.neuro.25.112701.142922, PMID: 12052908

[ref3] Andersen R. A. Essick G. K. Siegel R. M. (1987). Neurons of area 7 activated by both visual stimuli and oculomotor behavior. Exp. Brain Res. 67, 316–322. doi: 10.1007/BF00248552 3622691

[ref4] Barash S. Bracewell R. M. Fogassi L. Gnadt J. W. Andersen R. A. (1991). Saccade-related activity in the lateral intraparietal area. I. Temporal properties; comparison with area 7a. J. Neurophysiol. 66, 1095–1108. doi: 10.1152/jn.1991.66.3.1095, PMID: 1753276

[ref5] Battaglia-Mayer A. Caminiti R. (2018). Parieto-frontal networks for eye–hand coordination and movements. Handb Clin Neurol 151, 499–524. doi: 10.1016/B978-0-444-63622-5.00026-7 29519477

[ref6] Bisley J. W. Goldberg M. E. (2010). Attention, intention, and priority in the parietal lobe. Annu. Rev. Neurosci. 33, 1–21. doi: 10.1146/annurev-neuro-060909-152823, PMID: 20192813 PMC3683564

[ref7] Blatt G. J. Andersen R. A. Stoner G. R. (1990). Visual receptive field organization and cortico-cortical connections of the lateral intraparietal area (area LIP) in the macaque. J. Comp. Neurol. 299, 421–445. doi: 10.1002/cne.902990404 2243159

[ref8] Brown J. W. Hanes D. P. Schall J. D. Stuphorn V. (2008). Relation of frontal eye field activity to saccade initiation during a countermanding task. Exp. Brain Res. 190, 135–151. doi: 10.1007/s00221-008-1455-0, PMID: 18604527 PMC2748998

[ref9] Butler A. B. Hodos W. (2005). Comparative vertebrate neuroanatomy: evolution and adaptation. New-York: Wiley-Liss.

[ref10] Caminiti R. Borra E. Visco-Comandini F. Battaglia-Mayer A. Averbeck B. B. Luppino G. (2017). Computational architecture of the parieto-frontal network underlying cognitive-motor control in monkeys. eNeuro 4, ENEURO.0306–ENEU16.2017. doi: 10.1523/ENEURO.0306-16.2017, PMID: 28275714 PMC5329620

[ref11] Caminiti R. Chafee M. V. Battaglia-Mayer A. Averbeck B. B. Crowe D. A. Georgopoulos A. P. (2010). Understanding the parietal lobe syndrome from a neurophysiological and evolutionary perspective. Eur. J. Neurosci. 31, 2320–2340. doi: 10.1111/j.1460-9568.2010.07291.x, PMID: 20550568 PMC2900452

[ref12] Caminiti R. Innocenti G. M. Battaglia-Mayer A. (2015). Organization and evolution of parieto-frontal processing streams in macaque monkeys and humans. Neurosci. Biobehav. Rev. 56, 73–96. doi: 10.1016/j.neubiorev.2015.06.014, PMID: 26112130

[ref13] Cavanaugh J. Berman R. A. Joiner W. M. Wurtz R. H. (2016). Saccadic corollary discharge underlies stable visual perception. J. Neurosci. 36, 31–42. doi: 10.1523/JNEUROSCI.2054-15.2016, PMID: 26740647 PMC4701964

[ref14] Churchland A. K. Kiani R. Shadlen M. N. (2008). Decision-making with multiple alternatives. Nat. Neurosci. 11, 693–702. doi: 10.1038/nn.2123, PMID: 18488024 PMC2453226

[ref9001] Collins C. E. Lyon D. C. Kaas J. H. (2005). Distribution across cortical areas of neurons projecting to the superior colliculus in new world monkeys. Comparative Study Anat. Rec. A Discov. Mol. Cell Evol. Biol. 285, 619–627. doi: 10.1002/ar.a.20207 15912524

[ref15] Constantin A. G. Wang H. Monteon J. A. Martinez-Trujillo J. C. Crawford J. D. (2009). 3-dimensional eye-head coordination in gaze shifts evoked during stimulation of the lateral intraparietal cortex. Neuroscience 164, 1284–1302. doi: 10.1016/j.neuroscience.2009.08.066, PMID: 19733631

[ref16] Cooke D. F. Taylor C. S. R. Moore T. Graziano M. S. A. (2003). Complex movements evoked by microstimulation of the ventral intraparietal area. Proc. Natl. Acad. Sci. U. S. A. 100, 6163–6168. doi: 10.1073/pnas.1031751100, PMID: 12719522 PMC156343

[ref17] Crapse T. B. Sommer M. A. (2008). Corollary discharge circuits in the primate brain. Curr. Opin. Neurobiol. 18, 552–557. doi: 10.1016/j.conb.2008.09.017 18848626 PMC2702467

[ref18] Curtis C. E. Cole M. W. Rao V. Y. D’Esposito M. (2005). Canceling planned action: an fMRI study of countermanding saccades. Cereb. Cortex 15, 1281–1289. doi: 10.1093/cercor/bhi011, PMID: 15616130

[ref9002] Desmurget M. Richard N. Beuriat P. A. Szathmari A. Mottolese C. Duhamel J. R.. (2018). Selective inhibition of volitional hand movements after stimulation of the dorsoposterior parietal cortex in humans. Curr. Biol. 28, 3303–3309. doi: 10.1016/j.cub.2018.08.027 30318348

[ref19] Duhamel J. R. Colby C. L. Goldberg M. E. (1992). The updating of the representation of visual space in parietal cortex by intended eye movements. Science 255, 90–92. doi: 10.1126/science.1553535 1553535

[ref20] Emeric E. E. Brown J. W. Boucher L. Carpenter R. H. S. Hanes D. P. Harris R.. (2007). Influence of history on saccade countermanding performance in humans and macaque monkeys. Vis. Res. 47, 35–49. doi: 10.1016/j.visres.2006.08.032, PMID: 17081584 PMC1815391

[ref21] Ferraina S. Johnson P. B. Garasto M. R. Battaglia-Mayer A. Ercolani L. Bianchi L.. (1997). Combination of hand and gaze signals during reaching: activity in parietal area 7 m of the monkey. J. Neurophysiol. 77, 1034–1038. doi: 10.1152/jn.1997.77.2.1034, PMID: 9065868

[ref22] Ferraina S. Paré M. Wurtz R. H. (2002). Comparison of cortico-cortical and cortico-collicular signals for the generation of saccadic eye movements. J. Neurophysiol. 87, 845–858. doi: 10.1152/jn.00317.2001 11826051

[ref23] Ferrier D. (1875). Experiments on the brain of monkeys.–No. I. Proc. R. Soc. Lond. 23, 409–430. doi: 10.1098/rspl.1874.0058

[ref24] Foerster O. (1931). The cerebral cortex of man. Lancet 218, 309–312.

[ref25] Galletti C. Gamberini M. Kutz D. F. Fattori P. Luppino G. Matelli M. (2001). The cortical connections of area V6: an occipito-parietal network processing visual information. Eur. J. Neurosci. 13, 1572–1588. doi: 10.1046/j.0953-816x.2001.01538.x, PMID: 11328351

[ref26] Gardner E. P. Babu K. S. Reitzen S. D. Ghosh S. Brown A. S. Chen J.. (2007). Neurophysiology of Prehension. I. Posterior parietal cortex and object-oriented hand behaviors. J. Neurophysiol. 97, 387–406. doi: 10.1152/jn.00558.2006, PMID: 16971679 PMC2868366

[ref27] Gaymard B. Lynch J. Ploner C. J. Condy C. Rivaud-Péchoux S. (2003). The parieto-collicular pathway: anatomical location and contribution to saccade generation. Eur. J. Neurosci. 17, 1518–1526. doi: 10.1046/j.1460-9568.2003.02570.x, PMID: 12713655

[ref28] Goodale M. A. Milner A. D. (1992). Separate visual pathways for perception and action. Trends Neurosci. 15, 20–25. doi: 10.1016/0166-2236(92)90344-8 1374953

[ref29] Gottlieb J. Goldberg M. E. (1999). Activity of neurons in the lateral intraparietal area of the monkey during an antisaccade task. Nat. Neurosci. 2, 906–912. doi: 10.1038/13209, PMID: 10491612

[ref30] Hall N. J. Colby C. L. (2011). Remapping for visual stability. Philos. Trans. R. Soc. Lond. B 366, 528–539. doi: 10.1098/rstb.2010.0248 21242141 PMC3030834

[ref31] Hampshire A. Sharp D. J. (2015). Contrasting network and modular perspectives on inhibitory control. Trends Cogn. Sci. 19, 445–452. doi: 10.1016/j.tics.2015.06.006, PMID: 26160027

[ref32] Hanes D. P. Patterson W. F. Schall J. D. (1998). Role of frontal eye fields in countermanding saccades: visual, movement, and fixation activity. J. Neurophysiol. 79, 817–834. doi: 10.1152/jn.1998.79.2.817 9463444

[ref33] Hanes D. P. Schall J. D. (1996). Neural control of voluntary movement initiation. Science 274, 427–430. doi: 10.1126/science.274.5286.427 8832893

[ref34] Hanning N. M. Fernández A. Carrasco M. (2023). Dissociable roles of human frontal eye fields and early visual cortex in presaccadic attention. Nat. Commun. 14:5381. doi: 10.1038/s41467-023-40678-z, PMID: 37666805 PMC10477327

[ref35] Huk A. C. Katz L. N. Yates J. L. (2017). The role of the lateral intraparietal area in (the study of) decision making. Annu. Rev. Neurosci. doi: 10.1146/annurev-neuro-072116 PMC6289048 28772104

[ref36] Ito S. Stuphorn V. Brown J. W. Schall J. D. (2003). Performance monitoring by the anterior cingulate cortex during saccade countermanding. Science 302, 120–122. doi: 10.1126/science.1087847, PMID: 14526085

[ref37] Jarvstad A. Gilchrist I. D. (2019). Cognitive control of saccadic selection and inhibition from within the core cortical saccadic network. J. Neurosci. 39, 1419–1418. doi: 10.1523/JNEUROSCI.1419-18.2018, PMID: 30683684 PMC6435832

[ref38] Kaas J. H. (2008). The evolution of the complex sensory and motor systems of the human brain. Brain Res. Bull. 75, 384–390. doi: 10.1016/j.brainresbull.2007.10.009, PMID: 18331903 PMC2349093

[ref39] Kaas J. H. Gharbawie O. A. Stepniewska I. (2011). The organization and evolution of dorsal stream multisensory motor pathways in primates. Front. Neuroanat. 5:34. doi: 10.3389/fnana.2011.00034, PMID: 21716641 PMC3116136

[ref40] Kaas J. H. Qi H.-X. Stepniewska I. (2018). The evolution of parietal cortex in primates. Handb. Clin. Neurol. 151, 31–52. doi: 10.1016/B978-0-444-63622-5.00002-4 29519465

[ref41] Katz P. S. Harris-Warrick R. M. (1999). The evolution of neuronal circuits underlying species-specific behavior. Curr. Opin. Neurobiol. 9, 628–633. doi: 10.1016/S0959-4388(99)00012-4 10508740

[ref42] Kurylo D. D. Skavenski A. A. (1991). Eye movements elicited by electrical stimulation of area PG in the monkey. J. Neurophysiol. 65, 1243–1253. doi: 10.1152/jn.1991.65.6.1243, PMID: 1714952

[ref43] Lewis J. W. Van Essen D. C. (2000a). Corticocortical connections of visual, sensorimotor, and multimodal processing areas in the parietal lobe of the macaque monkey. J. Comp. Neurol. 428, 112–137. doi: 10.1002/1096-9861(20001204)428:1<112::AID-CNE8>3.0.CO;2-9, PMID: 11058227

[ref44] Lewis J. W. Van Essen D. C. (2000b). Mapping of architectonic subdivisions in the macaque monkey, with emphasis on parieto-occipital cortex. J. Comp. Neurol. 428, 79–111. doi: 10.1002/1096-9861(20001204)428:1<79::AID-CNE7>3.0.CO;2-Q, PMID: 11058226

[ref45] Li C.-S. R. Mazzoni P. Andersen R. A. (1999). Effect of reversible inactivation of macaque lateral intraparietal area on visual and memory saccades. J. Neurophysiol. 81, 1827–1838. doi: 10.1152/jn.1999.81.4.1827, PMID: 10200217

[ref46] Liu Y. Yttri E. A. Snyder L. H. (2010). Intention and attention: different functional roles for LIPd and LIPv. Nat. Neurosci. 13, 495–500. doi: 10.1038/nn.2496, PMID: 20190746 PMC2846989

[ref47] Lo C.-C. Boucher L. Pare M. Schall J. D. Wang X.-J. (2009). Proactive inhibitory control and attractor dynamics in countermanding action: a spiking neural circuit model. J. Neurosci. 29, 9059–9071. doi: 10.1523/JNEUROSCI.6164-08.2009, PMID: 19605643 PMC2756461

[ref48] Logan G. D. Cowan W. B. (1984). On the ability to inhibit thought and action: a theory of an act of control. Psychol. Rev. 91, 295–327. doi: 10.1037/0033-295X.91.3.295 24490789

[ref49] Lynch J. C. (1992). Saccade initiation and latency deficits after combined lesions of the frontal and posterior eye fields in monkeys. J. Neurophysiol. 68, 1913–1916. doi: 10.1152/jn.1992.68.5.1913, PMID: 1479455

[ref50] Lynch J. C. Graybiel A. M. Lobeck L. J. (1985). The differential projection of two cytoarchitectonic subregions of the inferior parietal lobule of macaque upon the deep layers of the superior colliculus. J. Comp. Neurol. 235, 241–254. doi: 10.1002/cne.902350207, PMID: 3998211

[ref51] Lynch J. C. McLaren J. W. (1989). Deficits of visual attention and saccadic eye movements after lesions of parietooccipital cortex in monkeys. J. Neurophysiol. 61, 74–90. doi: 10.1152/jn.1989.61.1.74 2918350

[ref52] Lynch J. C. Mountcastle V. B. Talbot W. H. Yin T. C. (1977). Parietal lobe mechanisms for directed visual attention. J. Neurophysiol. 40, 362–389. doi: 10.1152/jn.1977.40.2.362 403251

[ref53] Mariani O. S. C. Lima B. Soares J. G. M. Mayer A. Franca J. G. Gattass R. (2019). Partitioning of the primate intraparietal cortex based on connectivity pattern and immunohistochemistry for Cat-301 and SMI-32. J. Comp. Neurol. 527, 694–717. doi: 10.1002/cne.24438, PMID: 29577279

[ref54] Medalla M. Barbas H. (2006). Diversity of laminar connections linking periarcuate and lateral intraparietal areas depends on cortical structure. Eur. J. Neurosci. 23, 161–179. doi: 10.1111/j.1460-9568.2005.04522.x, PMID: 16420426

[ref55] Moore T. (1999). Shape representations and visual guidance of saccadic eye movements. Science 285, 1914–1917. doi: 10.1126/science.285.5435.1914 10489371

[ref56] Mountcastle V. B. Lynch J. C. Georgopoulos A. Sakata H. Acuna C. (1975). Posterior parietal association cortex of the monkey: command functions for operations within extra personal space. J. Neurophysiol. 38, 871–908. doi: 10.1152/jn.1975.38.4.871, PMID: 808592

[ref57] Mushiake H. Fujii N. Tanji J. (1999). Microstimulation of the lateral wall of the intraparietal sulcus compared with the frontal eye field during oculomotor tasks. J. Neurophysiol. 81, 1443–1448. doi: 10.1152/jn.1999.81.3.1443, PMID: 10085372

[ref58] Nakamura K. Colby C. L. (2000). Visual, saccade-related, and cognitive activation of single neurons in monkey extrastriate area V3A. J. Neurophysiol. 84, 677–692. doi: 10.1152/jn.2000.84.2.677 10938295

[ref59] Niu M. Impieri D. Rapan L. Funck T. Palomero-Gallagher N. Zilles K. (2020). Receptor-driven, multimodal mapping of cortical areas in the macaque monkey intraparietal sulcus. eLife 9:e55979. doi: 10.7554/eLife.55979, PMID: 32613942 PMC7365665

[ref60] Osada T. Ohta S. Ogawa A. Tanaka M. Suda A. Kamagata K.. (2019). An essential role of the intraparietal sulcus in response inhibition predicted by parcellation-based network. J. Neurosci. 39, 2509–2521. doi: 10.1523/JNEUROSCI.2244-18.2019, PMID: 30692225 PMC6435821

[ref61] Paré M. Dorris M. C. (2011). The role of posterior parietal cortex in the regulation of saccadic eye movements. Liversedge S. P. Gilchrist I. Everling S. (eds), The Oxford handbook of eye movements. Oxford: Oxford University Press

[ref62] Paré M. Hanes D. P. (2003). Controlled movement processing: superior colliculus activity associated with countermanded saccades. J. Neurosci. 23, 6480–6489. doi: 10.1523/JNEUROSCI.23-16-06480.2003 12878689 PMC6740637

[ref63] Paré M. Munoz D. P. (1996). Saccadic reaction time in the monkey: advanced preparation of oculomotor programs is primarily responsible for express saccades occurrence. J. Neurophysiol. 76, 3666–3681. doi: 10.1152/jn.1996.76.6.3666, PMID: 8985865

[ref64] Paré M. Wurtz R. H. (1997). Monkey posterior parietal cortex neurons antidromically activated from superior colliculus. J. Neurophysiol. 78, 3493–3497. doi: 10.1152/jn.1997.78.6.3493 9405568

[ref65] Paré M. Wurtz R. H. (2001). Progression in neuronal processing for saccadic eye movements from parietal cortex area lip to superior colliculus. J. Neurophysiol. 85, 2545–2562. doi: 10.1152/jn.2001.85.6.2545 11387400

[ref66] Park I. M. Meister M. L. R. Huk A. C. Pillow J. W. (2014). Encoding and decoding in parietal cortex during sensorimotor decision-making. Nat. Neurosci. 17, 1395–1403. doi: 10.1038/nn.3800, PMID: 25174005 PMC4176983

[ref67] Pouget P. Logan G. D. Palmeri T. J. Boucher L. Paré M. Schall J. D. (2011). Neural basis of adaptive response time adjustment during saccade countermanding. J. Neurosci. 31, 12604–12612. doi: 10.1523/JNEUROSCI.1868-11.2011, PMID: 21880921 PMC3173043

[ref68] Preuss T. M. (2007). “Evolutionary specializations of primate brain systems,” in Primate origins: adaptations and evolution. eds. Ravosa M. J. Dagosto M. (New York: Springer), 625–75.

[ref69] Rae C. L. Hughes L. E. Weaver C. Anderson M. C. Rowe J. B. (2014). Selection and stopping in voluntary action: a meta-analysis and combined fMRI study. NeuroImage 86, 381–391. doi: 10.1016/j.neuroimage.2013.10.012, PMID: 24128740 PMC3898966

[ref70] Rathelot J.-A. Dum R. P. Strick P. L. (2017). Posterior parietal cortex contains a command apparatus for hand movements. Proc. Natl. Acad. Sci. U.S.A. 114, 4255–4260. doi: 10.1073/pnas.1608132114 28373554 PMC5402465

[ref71] Roitman J. D. Shadlen M. N. (2002). Response of neurons in the lateral intraparietal area during a combined visual discrimination reaction time task. J. Neurosci. 22, 9475–9489. doi: 10.1523/JNEUROSCI.22-21-09475.2002 12417672 PMC6758024

[ref72] Ross C. F. Martin R. D. (2007). “The role of vision in the origin and evolution of primates,” in Evolution of nervous systems (Vol. 4): the evolution of primate nervous systems. eds. Kaas J. H. Preuss T. M. (Oxford: Elsevier), 59–78.

[ref73] Schall J. D. Morel A. King D. J. Bullier J. (1995). Topography of visual cortex connections with frontal eye field in macaque: convergence and segregation of processing streams. J. Neurosci. 15, 4464–4487. doi: 10.1523/JNEUROSCI.15-06-04464.1995 7540675 PMC6577698

[ref74] Schiller P. H. Tehovnic E. J. (2003). Cortical inhibitory circuits in eye-movement generation. Eur. J. Neurosci. 18, 3127–3133. doi: 10.1111/j.1460-9568.2003.03036.x 14656309

[ref75] Schiller P. H. Tehovnik E. J. (2001). Look and see: how the brain moves your eye about. Prog. Brain. Res. 134, 127–142. doi: 10.1016/S0079-6123(01)34010-4 11702539

[ref76] Schiller P. H. True S. D. Conway J. L. (1980). Deficits in eye movements following frontal eye-field and superior colliculus ablations. J. Neurophysiol. 44, 1175–1189. doi: 10.1152/jn.1980.44.6.1175, PMID: 6778974

[ref9005] Shen K. Paré M. (2006). Guidance of eye movements during visual conjunction search: local and global contextual effects on target discriminability. J. Neurophysiol. 95, 2845–2855. doi: 10.1152/jn.00898.2005 16467428

[ref77] Shibutani H. Sakata H. Hyvärinen J. (1984). Saccade and blinking evoked by microstimulation of the posterior parietal association cortex of the monkey. Exp. Brain Res. 55, 1–8. doi: 10.1007/BF00240493, PMID: 6745342

[ref78] Snyder L. H. Batista A. P. Andersen R. A. (1997). Coding of intention in the posterior parietal cortex. Nature 386, 167–170. doi: 10.1038/386167a0 9062187

[ref79] Stepniewska I. Fang P.-C. Kaas J. H. (2005). Microstimulation reveals specialized subregions for different complex movements in posterior parietal cortex of prosimian galagos. Proc. Natl. Acad. Sci. U. S. A. 102, 4878–4883. doi: 10.1073/pnas.0501048102, PMID: 15772167 PMC555725

[ref80] Strick P. L. Dum R. P. Rathelot J.-A. (2021). The cortical motor areas and the emergence of motor skills: a neuroanatomical perspective. Annu. Rev. Neurosci. 44, 425–447. doi: 10.1146/annurev-neuro-070918-050216, PMID: 33863253

[ref81] Stuphorn V. Brown J. W. Schall J. D. (2010). Role of supplementary eye field in saccade initiation: executive, not direct, control. J. Neurophysiol. 103, 801–816. doi: 10.1152/jn.00221.2009, PMID: 19939963 PMC2822692

[ref82] Stuphorn V. Schall J. D. (2006). Executive control of countermanding saccades by the supplementary eye field. Nat. Neurosci. 9, 925–931. doi: 10.1038/nn1714, PMID: 16732274

[ref83] Stuphorn V. Taylor T. L. Schall J. D. (2000). Performance monitoring by the supplementary eye field. Nature 408, 857–860. doi: 10.1038/35048576 11130724

[ref84] Supèr H. van der Togt C. Spekreijse H. Lamme V. A. F. (2004). Correspondence of presaccadic activity in the monkey primary visual cortex with saccadic eye movements. Proc. Natl. Acad. Sci. U. S. A. 101, 3230–3235. doi: 10.1073/pnas.0400433101 14970334 PMC365772

[ref85] Thier P. Andersen R. A. (1998). Electrical microstimulation distinguishes distinct saccade-related areas in the posterior parietal cortex. J. Neurophysiol. 80, 1713–1735. doi: 10.1152/jn.1998.80.4.1713, PMID: 9772234

[ref86] Thomas N. W. D. Paré M. (2007). Temporal processing of saccade targets in parietal cortex area LIP during visual search. J. Neurophysiol. 97, 942–947. doi: 10.1152/jn.00413.2006 17079346

[ref87] Tsotsos J. K. (2011). A computational perspective on visual attention. Cambridge: MIT Press.

[ref88] Ungerleider L. G. Galkin T. W. Desimone R. Gattass R. (2008). Cortical connections of area V4 in the macaque. Cereb. Cortex 18, 477–499. doi: 10.1093/cercor/bhm061 17548798

[ref89] Ungerleider L. G. Mishkin M. (1982). “Two cortical visual systems” in Analysis of visual behavior. eds. Ingle D. J. Goodale M. A. Mansfield R. J. W. (Cambridge, MA: MIT Press), 549–586.

[ref90] Vogt O. Vogt C. (1926). Die vergleichend-architektonische und vergleichend-reizphysiologische felderung der grobhirnrinde unter besonderer berücksichtigung der menschilchen. Naturwissenchaften 14, 1190–1194. doi: 10.1007/BF01451766

[ref91] Waitzman D. M. Ma T. P. Optican L. M. Wurtz R. H. (1991). Superior colliculus neurons mediate the dynamic characteristics of saccades. J. Neurophysiol. 66, 1716–1737. doi: 10.1152/jn.1991.66.5.1716, PMID: 1765803

[ref92] Wardak C. Olivier E. Duhamel J.-R. (2002). Saccadic target selection deficits after lateral intraparietal area inactivation in monkeys. J. Neurosci. 22, 9877–9884. doi: 10.1523/JNEUROSCI.22-22-09877.2002 12427844 PMC6757813

[ref93] Watson R. T. Valenstein E. Day A. Heilman K. M. (1994). Posterior neocortical systems subserving awareness and neglect. Neglect associated with superior temporal sulcus but not area 7 lesions. Arch. Neurol. 51, 1014–1021. doi: 10.1001/archneur.1994.00540220060015, PMID: 7944999

[ref94] Wilke M. Kagan I. Andersen R. A. (2012). Functional imaging reveals rapid reorganization of cortical activity after parietal inactivation in monkeys. Proc. Natl. Acad. Sci. U. S. A. 109, 8274–8279. doi: 10.1073/pnas.1204789109, PMID: 22562793 PMC3361455

[ref95] Wurtz R. H. (2008). Neuronal mechanisms of visual stability. Vis. Res. 48, 2070–2089. doi: 10.1016/j.visres.2008.03.021, PMID: 18513781 PMC2556215

[ref96] Xu K. Z. Anderson B. A. Emeric E. E. Sali A. W. Stuphorn V. Yantis S.. (2017). Neural basis of cognitive control over movement inhibition: human fMRI and primate electrophysiology evidence. Neuron 96, 1447–1458.e6. doi: 10.1016/j.neuron.2017.11.010, PMID: 29224723 PMC5747365

[ref97] Zhang Y. Fries P. (2023) Eccentricity-dependent saccadic reaction time: the roles of foveal magnification and attentional orienting. *bioRxiv*. Available at: 10.1101/2023.08.08.552339. [Epub ahead of preprint] PMC12281140 40697829

